# Supplementary material for: Anti-Cancer Properties of Two Intravenously Administrable Curcumin Formulations as Evaluated in the 3D Patient-Derived Cancer Spheroid Model
Source: Int J Mol Sci. 2024 Aug 5;25(15):8543. doi: 10.3390/ijms25158543 (PMC11313667; doi:10.3390/ijms25158543)
Supplement: Supplementary file 1 [file ijms-25-08543-s001.zip › ijms-3078215-supplementary.pdf]

Table S1. Modulation of standard mono- and double therapies through Curcumin and its formulations CurA and CurB. Negative values indicate inhibition of standard therapy. Cur, Curcumin overall; CurA, Curcumin A; CurB, Curcumin B; p-values calculated using Mann-Whitney-U-test.

	**Standard monotherapy**			**Standard double therapy**		
	Mean	Range	*p-value*	Mean	Range	*p-value*
Cur formulations	-0.03%	-37.05% – 24.00%	0.946	-1.21%	-9.40% – 25.00%	0.687
CurA	0.53%	-37.05% – 15.00%	0.724	3.33%	-4.34% – 25.00%	1.000
CurB	-0.53%	-26.70% – 24.00%	0.888	-6.51%	-9.40% – -2.98%	0.485

Table S2. Modulation of Curcumin and its formulations through Art, Res and VitC. Negative stimulation values correspond to inhibitory effects on Curcumin and its formulations. Cur, Curcumin overall; CurA, Curcumin A; CurB, Curcumin B; Art, Artesunate; Res, Resveratrol; VitC, Vitamin C; p-values calculated using Mann-Whitney-U-test.

	**Art**			**Res**			**VitC**		
	Mean	Range	*p-value*	Mean	Range	*p-value*	Mean	Range	*p-value*
Cur formulations	-7.81%	-38.90% – 31.07%	0.020	0.01%	-38.10% – 29.50%	0.869	-5.80%	-42.34% – 12.72%	0.149
CurA	-7.68%	-38.90% – 31.07%	0.102	2.17%	-24.10% – 29.50%	0.751	-5.45%	-42.34% – 8.99%	0.217
CurB	-8.04%	-18.30% – 6.50%	0.031	-6.85%	-38.10% – 13.54%	0.589	-6.85%	-30.80% – 12.72%	0.310
